# Supplementary material for: PANGEA: A New Gene Set Enrichment Tool for Drosophila and Common Research Organisms
Source: bioRxiv. 2023 Apr 17:2023.02.20.529262. Originally published 2023 Feb 21. Preprint. [Version 2] doi: 10.1101/2023.02.20.529262 (PMC9980003; doi:10.1101/2023.02.20.529262)

Supplementary Figure 1: The proteomic dataset of high-confident preys of four bait proteins (Fl(2)d, METTL14, METTL3, Nito) was obtained from the supplementary table 2 of the published study by Tang et al (PMID: 33649236, https://www.ncbi.nlm.nih.gov/pmc/articles/PMC7958400/). PANGEA "Search Multiple" function was used and Phenotype annotation was selected. The comparison of the enrichment of *Drosophila* phenotype annotation from the interacting proteins of four different baits was illustrated using a heatmap.

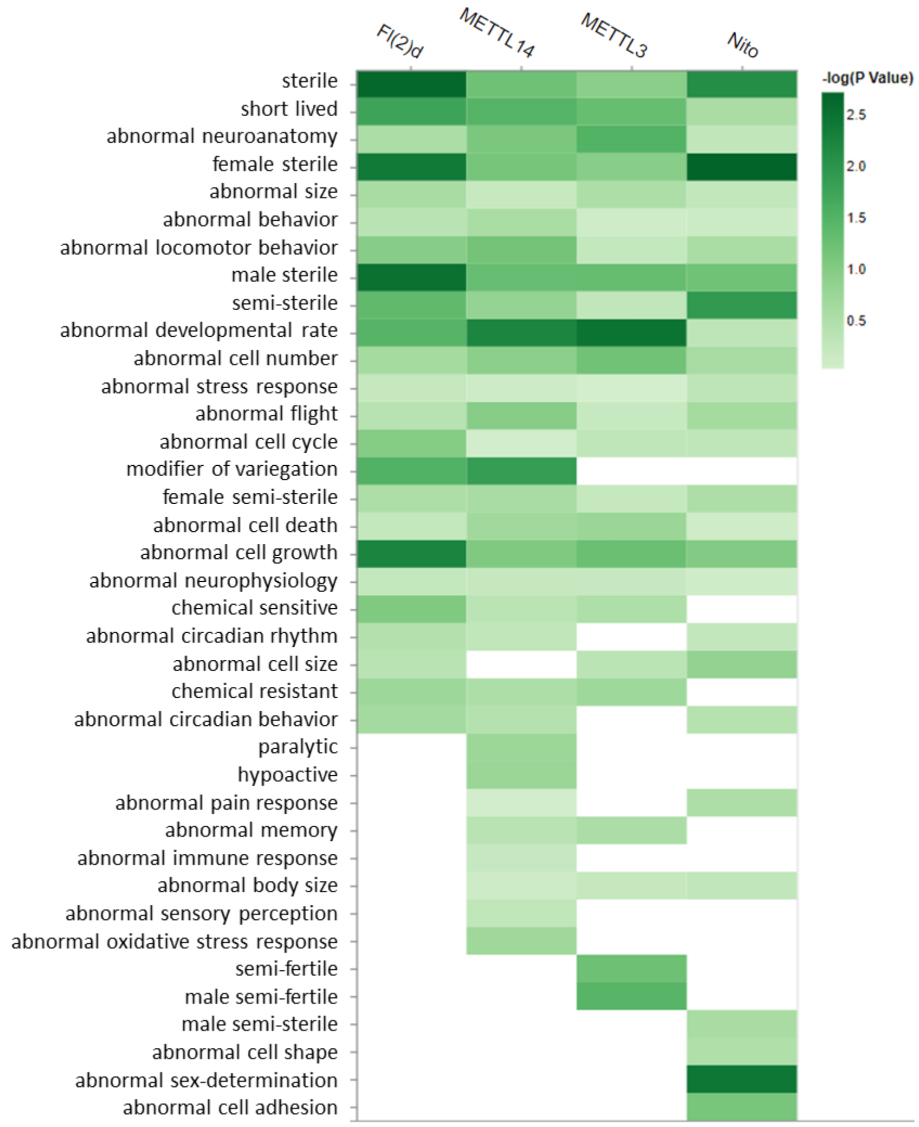

Supplement: Supplement 1 [file media-1.pdf]
